# Residence registration to cope with homelessness: evidence from a qualitative research study in Milan

**DOI:** 10.1186/s41118-021-00147-1

**Published:** 2021-12-18

**Authors:** M. Pasqualini, G. Bazzani

**Affiliations:** 1 Observatoire Sociologique du Changement (OSC) Sciences Po, Paris & IUSSP CRVS, Paris, France; 2 Department of Political and Social Sciences, University of Florence, Florence, Italy

## Abstract

Homeless people are one of the most vulnerable and marginalized groups in developed countries, and their homelessness situation often persists over the long term. However, so far, no studies have explained the specific role played by residence registration as it relates to deprivation amongst the homeless population and its contribution to improving the lives of homeless people. This paper investigates the paths homeless people in Milan use to access residence registration, via a case study in the city of Milan. Home to Italy’s largest homeless population, the city of Milan has implemented the innovative *ResidenzaMi* project to improve access to residence registration for homeless people. The study considers official statistics and individual interviews with service providers involved in the registration process. It further investigates the main factors impeding the registration process and outlines the consequences of the COVID-19 pandemic. Results from our study indicate that a residence certificate plays a critical role in helping homeless people exercise their rights and access the services they need to escape homelessness. Our findings suggest the importance of a holistic, multidimensional approach to ensure access to residence registration for homeless persons.

## Introduction

Homeless people are among the most vulnerable and marginalized subpopulation in developed countries (Fowler et al., 2019; Lee et al., 2010; Meanwell, 2012). However, little is known about the socio-demographic characteristics of the homeless population and its access to residence registration in Italy, despite it bearing on the homeless population's quality of life. Residence registration allows people in Italy to access services, obtain goods, and exercise their rights. Countries requiring residence registration aim to achieve complete registration but are hindered by the under-registration of marginalized populations, such as foreign nationals and homeless people.

Homelessness is not simply a severe lack of material resources. It also includes a lack of opportunities for a person to develop their own capabilities (Alkire, 2013; Sen, 1985). The concept of capability as a human right (Nussbaum, 2009) has been used to understand homelessness. Housing is a fundamental tool in maintaining a fair level of dignity, stability, and privacy. It also provides the opportunity and the freedom to pursue self-actualization (O’Shaughnessy et al., 2020). Homelessness is tied to deep social inequalities, illiteracy, low life expectancy, poor health conditions, and almost non-existent social and emotional networks (Batterham, 2019; Decembrotto, 2017; LaGory et al., 2001; Lee et al., 2010; Meanwell, 2012). The scarcity of official data on the number of homeless people does not allow us to provide a precise number. However, according to the last national survey on extreme poverty in Italy (ISTAT, 2015), homeless people comprise about 2.43 per thousand of the Italian population living in the 158 municipalities involved in the study. One-third of them do not appear in official statistics since they do not have a residential address (ISTAT, 2015). Home to the greatest number of homeless people in Italy (Feantsa, 2018), Milan offers the most advanced services to support residence registration for homeless people: the *ResidenzaMi* project.

This project shows how a pathway to escape homelessness requires access to basic rights and opportunities, which may require social and economic assistance for people experiencing extreme hardship. However, to access many forms of social and economic assistance in Italy, people need a proof of residence registration. To obtain this proof, people also need proof of legal identity. People who become homeless often do not have or have lost their residence registration. People who are deemed unavailable or not living at the residence address are removed from the Italian Residence Population Registry. Civil offices manage this long and complex process, which involves numerous checks. According to Italian law, when a person is not recorded in the Residence Population Registry, they can lose important social, political, and civil rights, which contributes to their hardship (Gnocchi, 2009). This study aims to contribute to existing literature by addressing important aspects related to the different paths homeless people use to access residence registration. Considering the different strategies for coping with homelessness, our hypothesis is that a residence certificate is critical to allowing homeless people to access public services. The lack of a residence certificate prevents homeless people from accessing services beyond their most basic needs. A residence certificate lets people join the labour market, sign a housing rental agreement, access health services beyond emergency situations, and access social services, as needed. It is a fundamental, constitutionally guaranteed right that may offer homeless people opportunities to improve their living conditions. In short, a residence certificate is the key to a better life for homeless people.

This research aims to contribute to current knowledge about the different paths, personal and structural obstacles, and enabling factors involved in the residence registration process for homeless people in Italy through a support service provider lens. The characteristics of the homeless population in Milan are described with quantitative data drawn from registers and a national survey. Semi-structured interviews were conducted with five support service providers identified by the Milan municipal office responsible for the residence registration of homeless people. Participants were asked to describe their work methods and the paths homeless people use to access residence registration.

## Background

According to the last national survey on the condition of persons living in extreme poverty (ISTAT, 2015), the estimated number of homeless persons using aid services in Italy was about 50,724. Approximately 24% of homeless persons were accommodated in Milan (i.e. about 12,022 individuals).

In Italy, an estimated one third of homeless are not legally registered (ISTAT, 2015; Table 1, column a).^1^ In 2014, women made up about 15% of the Italian homeless population; about 55% of homeless people were foreign nationals, mostly from non-European Union (EU) countries (Table 1, column a). Half of the Italian homeless population is between 35 and 54 years old, and most live in northern Italy (58.8 percent).

**Table 1:** Socio-demographic characteristics of the homeless using services in Italy

Variables	Italy		
	(a)	(b)	(c)
	2014		
	All; *n* (%)	Without residence certificate; *n* (%)	With residence certificate; *n* (%)
*N*	4726	1304	3391
Gender			
Women	729 (15.4)	195 (14.9)	530 (15.6)
Country of birth			
Italy	2112 (44.7)	65 (5)	2041 (60.2)
Other			
European Union	643 (13.6)	304 (23.3)	334 (9.9)
North Africa	622 (13.1)	217 (16.6)	405 (11.9)
Sub-Saharan Africa	528 (11.1)	270 (20.7)	253 (7.5)
South America	103 (2.2)	51 (3.9)	50 (1.5)
Middle East	138 (3)	73 (5.6)	61 (1.8)
Other	580 (12.3)	324 (24.9)	247 (7.2)
Age groups^a^			
< 18	N/A	N/A	N/A
18–34	1191 (25.2)	580 (44.5)	603 (17.8)
35–54	2363 (50)	577 (44.2)	1774 (52.3)
55–64	890 (18.8)	118 (9.1)	764 (22.5)
65+	282 (6.0)	29 (2.2)	250 (7.4)
Geographic location			
North	2779 (58.8)	639 (49)	2121 (62.5)
South	1107 (23.4)	438 (33.6)	664 (19.6)
Center	840 (17.8)	227 (17.4)	606 (17.9)
Without residence certificate^b^	1304 (27.7)		
Source	ISTAT		

ISTAT data relate to the Detection of Extreme Poverty (Year 2014) survey. Processing was carried out at the ISTAT Laboratory for the Analysis of Elementary Data in compliance with legislation on statistical law enforcement and the protection of personal data. The survey was conducted to investigate the living conditions of people in extreme poverty. The survey covered all individuals who, living in the street, had used at least one free aid service such as a social canteen or a dormitory in one of the 158 municipalities selected on the basis of their demographic dimension (more than 30,000 inhabitants). Children, citizens of Rome, women living in protected housing for domestic violence, and people living in smaller municipalities were excluded from the survey

fRDB data relate to a point-in-time count sampling procedure conducted in Milan in 2018 by Fondazione Rodolfo De Benedetti and Bocconi University. This sampling provides a picture of the homeless population living in Milan by collecting information on gender, age, and citizenship status in a single night

n.d., Observations less than 10 cannot be reported to protect personal data

N/A, not available

^a^Data for 2013 are available only for homeless interviewed in dormitories (source: fRDB). The age class is slightly different from other sources/years and is broken down as follows: > 25; 25‒34; 35‒44; 45‒60; 60+

^b^In the ISTAT survey, the question related to the residence certificate presents 31 missing answers in the Italy sample and 10 in the Milan subsample

The number of observations for 2003, 2008, and 2018 have been calculated by multiplying the total with the percentage published by fRDB

According to national representative data, individuals without a residence certificate are significantly younger compared with the general homeless population (Table 1, column b). In addition, homeless people without a residence certificate are almost all foreign nationals, many from non-EU countries.

### Contributing factors

#### The nature of homeless experience and its consequences

Existing research has shown that the amount of time people spend homeless deeply characterizes their life path and their likelihood of escaping homelessness (Batterham, 2019). More specifically, homelessness can be differentiated as one of the following (Culhane & Kuhn, 1998; Lee et al., 2010):

- Transitory or temporary: this refers to a short, extraordinary experience of housing deprivation in a person’s life course, or once in their lifetime.
- Episodic: this describes a cyclical experience of housing deprivation characterized by a series of entries and exits from homelessness. This could refer to a vast range of different experiences; for example, people who are homeless for certain periods and other times live in prison, treatment centres, or low-income housing (Kuhn & Culhane, 1998).
- Chronic: this is typically the traditional case of homelessness (Lee et al., 2010). By nature, it is less visible and has the lowest probability of being resolved.

#### Personal deficits and structural factors

Existing literature on the main risk factors associated with homelessness is strongly polarized around the explanations of personal deficit vs. structural dynamics (Formentin et al., 2009; Lee et al., 2010). While studies exploring personal deficit mainly focus on drugs, addiction, or mental health issues (Caton et al., 1994; Caton et al., 1995; LaGory et al., 2001; McQuistion et al., 2014), structural dynamics focus on socio-economic and cultural factors, such as the inter-generational transmission of poverty and the impact of economic crises or other external events on homelessness.

However, homelessness is rarely caused by a single, specific factor. Rather, it is usually due to a cumulation of events over a person’s life course that might cause impoverishing paths linked to disease, addiction, and marital disruption (Lee et al., 2010; Meo, 2009; Shlay & Rossi, 1992). For example, drawing from the first representative survey in Europe among homeless people, Braga and Corno showed that unemployment was the main reason for homelessness in Italy when combined with breakdowns in family relationships (Braga & Corno, 2011).

### Approaches to combatting homelessness

There are several approaches designed to help homeless people (Ministero del Lavoro e delle Politiche Sociali, 2015). These include:

- Residual services, which provide last resort services designed to fill in gaps that cannot otherwise be addressed by society. These services address a person’s basic needs, such as food, clothes, showers, and dormitories for emergencies that occur in colder months. Residual services are unstructured by nature, as they are designed to deal with emergencies.
- Low-threshold services, which include services that help reduce harm to people, make assistance easily accessible, and minimize or remove barriers for people to access help or care (Rhodes & Hedrich, 2010). Low-threshold services are typically used in public health services to deal with drug and alcohol addiction (Marlatt & Witkiewitz, 2002; Melles et al., 2007; Ministero del Lavoro e delle Politiche Sociali, 2015). In the case of homelessness, although these primary services are designed to help homeless people meet their primary needs (i.e. food, dormitories, medicine, etc.), they also offer dignified survival conditions to help homeless people access subsequent social assistance.
- Staircase interventions, which use a succession of measures that start with reception and extend to social rehabilitation. This approach helps empower people by setting a progressive plan to gain or regain social skills and meet other needs such as finding housing or getting a job, among others.
- Holistic or multidimensional interventions, which use a variety of services to cover different types of needs with different levels of intensity. Unlike the staircase approach, it is not based on a progressive series of standardized steps. Instead, this approach is based on a personal relationship with a service provider who helps identify personal objectives to lead a person to social inclusion. This type of intervention includes housing-led and housing first approaches (Aubry et al., 2017; Cortese, 2017; Tsemberis & Eisenberg, 2000; Tsemberis et al., 2004).

Among these different approaches, it is broadly recognized that beyond residual and emergency-oriented interventions, a person’s independence, wellbeing, and social integration can be reached using an individual path to help them meet their specific needs and assume control of their own life. Thus, once a person has received first aid services and their basic needs are met, it is essential that they desire change. This might be done through community processes to help deal with social stigma and help people develop skills such as interpersonal communication, self-motivation, and problem-solving (Anderson et al., 1994; Branca & Colombo, 2003; Goffman, 1963).

## Residence registration for homeless people in Italy

In Italy, most social interventions adopted to fight homelessness are services that might be both temporary or stable over time and use a residual approach. The staircase approach was born in the early 1960s in the United States, but was adopted in Italy in the late 1970s as an experimental way to rehabilitate patients with severe mental disorders (*Law 180/1978 Legge Basaglia*, also known as the Italian Mental Health Act). However, further institutionalization of this approach has led to excessively standardized and homologating practices (Ministero del Lavoro e delle Politiche Sociali, 2015).

The holistic approach spread in Italy following experiences in the United Kingdom and the U.S. (Tsemberis & Eisenberg, 2000) empirically showing that independent housing and reintegration into the labour market through community work can lead homeless people out of their condition of severe marginalization. These practices are based on recognizing the relevance of a home not only as a right, but as a person’s starting point on their path towards better life conditions (Ministero del Lavoro e delle Politiche Sociali, 2015).

### The importance of a residence certificate

Within the staircase and multidimensional approaches, residence registration is key to improving the living conditions of homeless people in countries where residence registration is required.^2^ Living without a residence certificate means that homeless people cannot access the services and social benefits to which they are entitled. It forces homeless people to adapt their lives to irregularity and instability, which makes it even harder to cope with situations of extreme poverty and need.

For example, residence registration is a prerequisite for Municipalities to provide national identity (ID) card^3^ that is also a proof of legal identity (Avvocato di Strada, 2019). Indeed, although birth and marriage certificates are useful documents to help authorities in the individuals’ identification and in the request for residence permit, they cannot substitute the residence certificate and the ID card.^4^

Essentially, people without a residence certificate have limited access to the Italy’s national social and health systems. Thus, they cannot:

- Have a family doctor;
- Access the hospital (except possibly for first aid);
- Have official identification documents (such as, the ID card, the driver’s license, the health insurance card or the electoral card);
- Be registered for employment services;
- Access aid from social services; or
- Renew their residence permit, in the case of foreign nationals.

However, legislation recognizes that registration cannot be denied to homeless people, since “…residence registration is a subjective right (and not a concession), it is recognized by the Italian legal system (*Law No. 1228 of 24.12.1954*, or the Registry Law) to all citizens, with the exception of foreign nationals not regularly residing in the area” (Federazione Italiana Organismi per le Persone Senza Dimora, n.d.). Italian law also provides specific requirements for foreign nationals depending on whether or not they are EU citizens. That is, European citizens have the right to stay in Italy for a period of less than 3 months without any formalities. If they want to stay longer than three months, they must register for a residence certificate. To do this, they must present a valid passport or identity document and documentation that proves they:

- Are employed or self-employed;
- Have enough economic resources;
- Are a student; or
- Are a family member living or reuniting with a European citizen who has the right to reside in Italy.

The process for registering foreign nationals from outside the EU with a legal residence permit is the same as residence registration for Italian citizens.^5^ Although adults need to renew their ID cards every 10 years, residence registration does not need to be renewed. If a person moves to a different Italian city, they must follow the same process to obtain a new residence registration.

### Registration options for Italy’s homeless population

At the local level across Italy, a government official records the position of all the individuals who have established their home in each municipality. Italy’s residence registration system allows homeless people to establish a residence:

- In their individual home, in the municipality where they currently live, or in the municipality where they were born (DPR. 223 of 30.05.1989); or
- Using a fictitious street that is not territorially existent but equivalent in legal value (ISTAT Circular no. 29/1992).^6^

The ability to designate a fictitious street as a residence is important for people without a home, as it may allow residence registration for homeless people. To allow people with no home of their own to specify an address and obtain a residence certificate, some Italian municipalities provide a virtual address that does not exist at the territorial level, but which has legal value. Although this system might represent a solution to guarantee rights and services to homeless people, it is not standardized throughout Italy and strongly depends on individual municipalities. Of the more than 8000 municipalities in Italy, only 222 have recognized the existence of fictitious streets to allow residence registration for homeless people (Federazione Italiana Organismi per le Persone Senza Dimora, n.d.).

### Case study: Milan’s response to homelessness

Milan has the highest absolute homeless population in Italy, with approximately 2608 individuals, or 0.19% of its total population (Fondazione Rodolfo De Benedetti, 2018). The main characteristic of the homeless population in Milan is that the majority of them are service users, as most homeless people stay in dormitories.^7^ The prevalence of those living in the streets is residual because even if there are many homeless, the system can offer services to everyone.

According to 2018 census data on Milan’s homeless population collected by Fondazione Rodolfo De Benedetti, homeless women represent about 14.5% of the homeless population (Table 2, column f) and this number is consistent over time.^8^ The prevalence of foreign nationals among Milan’s homeless population has increased over time (55.6% in 2008 vs. 73 percent in 2018).^9^

**Table 2:** Socio-demographic characteristics of the homeless using services in Milan

Variables	Milan					
	(a)	(b)	(c)	(d)	(e)	(f)
	2008	2013	2014			2018
	All; *n* (%)	All; *n* (%)	All; *n* (%)	Without residence certificate; *n* (%)	With residence certificate; *n* (%)	All; *n* (%)
*N*	1152	2106	825	216	599	2608
Gender						
Women	164 (14.2)	299 (14.2)	101 (12.2)	22 (10.2)	77 (12.8)	378 (14.5)
Country of birth						
Italy	511 (44.4)	478 (22.7)	261 (31.6)	n.d	254 (42.2)	704 (27)
Other	641 (55.6)	1628 (77.3)				1904 (73)
European Union	N/A	N/A	156 (18.9)	64 (29.6)	91 (15.2)	N/A
North Africa	N/A	N/A	131 (15.9)	45 (20.8)	86 (14.4)	N/A
Sub-Saharan Africa	N/A	N/A	114 (13.8)	32 (14.8)	80 (13.4)	N/A
South America	N/A	N/A	46 (5.6)	18 (8.3)	26 (4.4)	N/A
Middle East	N/A	N/A	20 (2.4)	n.d	11 (1.9)	N/A
Other	N/A	N/A	97 (11.8)	35 (16.2)	51 (8.5)	N/A
Age groups^a^						
< 18	N/A	297 (14.1)	N/A	N/A	N/A	17 (1)
18–34	N/A	519 (24.6)	249 (30.2)	91 (42.1)	154 (25.7)	600 (23)
35–54	N/A	454 (21.6)	365 (44.2)	97 (44.9)	265 (44.3)	1390 (53)
55–64	N/A	628 (29.8)	142 (17.2)	28 (13)	120 (20.0)	418 (16)
65+	N/A	208 (9.9)	69 (8.4)	n.d	60 (10.0)	183 (7)
Without residence certificate^b^	N/A	637 (30.24)	216 (26.18)			1017 (39.00)
Source	fRDB	fRDB	ISTAT			fRDB

ISTAT data relate to the Detection of Extreme Poverty (Year 2014) survey. Processing was carried out at the ISTAT Laboratory for the Analysis of Elementary Data in compliance with legislation on statistical law enforcement and the protection of personal data. The survey was conducted to investigate the living conditions of people in extreme poverty. The survey covered all individuals who, living in the street, had used at least one free aid service such as a social canteen or a dormitory in one of the 158 municipalities selected on the basis of their demographic dimension (more than 30,000 inhabitants). Children, citizens of Rome, women living in protected housing for domestic violence, and people living in smaller municipalities were excluded from the survey

fRDB data relate to a point-in-time count sampling procedure conducted in Milan in 2018 by Fondazione Rodolfo De Benedetti and Bocconi University. This sampling provides a picture of the homeless population living in Milan by collecting information on gender, age, and citizenship status in a single night

Notice that only data coming from the same source can be compared. Namely, those from fRDB 2008, 2013 and 2018

n.d., Observations less than 10 cannot be reported to protect personal data

N/A, not available

^a^Data for 2013 are available only for homeless interviewed in dormitories (source: fRDB). The age class is slightly different from other sources/years and is broken down as follows: > 25; 25‒34; 35‒44; 45‒60; 60+

^b^In the ISTAT survey, the question related to the residence certificate presents 31 missing answers in the Italy sample and 10 in the Milan subsample

The number of observations for 2003, 2008, and 2018 have been calculated by multiplying the total with the percentage published by fRDB

In 2018, approximately 40 percent of Milan’s homeless population did not have a residence certificate. This prevalence is slightly higher compared to results from the ISTAT survey in 2014 (26 percent), and the same census data in 2013 (30 percent). The demographic characteristics of homeless people without a residence certificate living in Milan are representative of the same subgroup of the Italian homeless population, although significantly different from those of the overall homeless population (Table 2, column d).

In 2018, the City of Milan spent 8 million Euros in funding from the Cohesion Fund, under the European Structural and Investment Funds, to offer new public benefits to the homeless population,^10^ including:

- Help services;
- Dormitories;
- Orientation services;
- Educational services;
- Public showers; and
- 24-h listening centres.

### ResidenzaMi

As part of the changes enabled through this funding, the municipality of Milan assumed responsibility for promoting the registration process among the homeless population at municipal offices through a specific project called *ResidenzaMi* (formally translated as residence-me, with “Mi” doubling as the abbreviation for Milan). This service aims to guarantee residence registration for homeless people living in the municipalities of Milan.

In addition to providing new benefits, *ResidenzaMi* centralized and institutionalized the process of residence registration for homeless people. The initiative was also supported by a social action committee called Thieving Misery.^11^

Before 2019, homeless people could only obtain a residence certificate through social private, parishes, NGOs, and low-threshold services and organizations, referred to as the third sector, that autonomously and arbitrarily allowed homeless people to use the legal residence of these organizations if they were known to the groups and one of the organizations could confirm that the homeless person lived in the city. More specifically, parishes, NGOs, social services, and low-threshold services allowed vulnerable individuals who were using their services to use their address to obtain a residence certificate. This included helping foreign nationals renew their residence permit or providing an address for children who were unable live with their parents.^12^ Thus, before 2019 the process of obtaining a residence certificate was left to chance or depended on the availability and willingness of each organization.

The purpose of *ResidenzaMi* was to give homeless people access to a fictitious residence and institutionalize the process by strengthening and standardizing the way the city of Milan recognizes the legal rights of homeless people to a residence certificate. The choice to institutionalize this process and make its administration public was led by a desire to find a stable, less formal way to register homeless people*.* More specifically, the project used a joint participation tool to make municipal workers aware of the issue faced by homeless people and engage them in developing a solution. As a result, service counters were successfully opened in nine municipal offices throughout the city with the specific purpose of registering homeless people using each office’s address.^13^ At each counter, there are educators who receive homeless people, prepare their request documents, and begin the bureaucratic process through the Residence Population Registry.^14^

### Registration process

The first step of this process is called the *servizio filtro*, or screening service, which helps homeless people prepare their request for a residence certificate. Staff work with homeless people to collect their personal data, help fill out the residence request, and find and present the necessary documents. The screening service operates at easily identifiable institutional settings, often municipal offices. It is exclusively available to homeless people who live permanently in the territory of the Municipality of Milan; maintain an ongoing relationship with the territory in terms of interests, relationships, and affections; and who express the desire and intention to remain in the municipality. These criteria are confirmed using an introductory report or referral from the third sector, the *Servizio Sociale Professionale Territoriale* (SSPT) or Territorial Professional Social Services*,* basic health services, or specialists.^15^ This step is particularly relevant as it ensures that people are eligible to submit a request.

If foreign nationals are eligible to receive a residence certificate under Italian law, *ResidenzaMi* facilitates legal access, assists with collecting documents, and helps prepare the request. This service is particularly useful to foreign nationals, as it breaks down language and bureaucratic barriers. Furthermore, since many foreign nationals hold irregular or informal leases, *ResidenzaMi* helps them get a residence certificate using their actual address.

Once the residence certificate is obtained, homeless people can ask for the ID card at the City Hall, following the standard national procedure, and are eligible for all the other benefits come with it.^16^

## Methods

### Data collected

The objective of this study was to examine important aspects related to the different paths homeless people use to access residence registration in Italy, and to shed light on the critical role that a residence certificate plays in allowing homeless people to access public services and exercise their individual rights. We aimed to supplement those findings by conducting an exploratory analysis using qualitative interviews with service providers of *ResidenzaMi* to examine the paths homeless people use and the barriers they face in obtaining a residence certificate through service providers.

The only quantitative data available for those who applied for a residence certificate was drawn from the *ResidenzaMi* register*.* Since the data collected are sensitive, the only information available was related to aggregate characteristics about the types of applicants, such as gender, age, etc. (Table 3). Most applications received in 2019 were from men (70 percent), and more specifically from single men (68 percent). They were followed by single women at 16.8 percent. This finding is not surprising, since the share of homeless single men is the highest in the overall homeless population.^17^ Couples without dependent children made up only about 2 percent of total applications, while couples with minor children represented 5 percent, and single mothers with minor children represented about 8 percent in 2019. Moreover, approximately one-quarter of total applications in 2019 were made by individuals aged 40‒49. Finally, about 35.6 percent of those who applied for a residence certificate were Italian. Among foreign nationals, the most widely represented group were North Africans at about 24 percent. Thus, descriptive statistics show that the profile of applicants aligns with the general homeless population.

**Table 3:** Socio-demographic characteristics of homeless people in Milan who applied for a residence certificate through ResidenzaMi, 2019

Variables	*n* (%)
Gender	
Women	645 (30.0)
Age	
> 19	57 (2.9)
20‒29	332 (16.8)
30‒39	392 (19.8)
40‒49	491 (24.8)
50‒59	431 (21.8)
60‒69	208 (10.5)
70‒79	47 (2.3)
80+	11 (0.5)
Missing	13 (0.6)
Family structure	
Men and women with minor children	101 (5.1)
Single men with minor children	1 (1.0)
Women alone with minor children	161 (8.1)
Men and women	31 (1.6)
Single men with adult children	1 (0.0)
Single women with adult children	3 (0.1)
Single men	1,351 (68.2)
Single women	333 (16.8)
Country of birth	
Italy	706 (35.6)
North Africa	483 (24.4)
Sub-Saharan Africa	277 (14)
South America	143 (7.2)
Middle East	101 (5.0)
EU	170 (8.6)
Other	102 (5.2)

Source: *ResidenzaMi* register (2019). *N* = 1982

### Research methods

As there are no studies on the relationship between residence registration and homelessness, we undertook an exploratory analysis using qualitative interviews with support service providers to describe the characteristics of the homeless population and their access paths to residence registration (Creswell and Plano Clark 2010). National surveys and local registers do not contain information regarding the residence registration process other than whether respondents were or were not registered. For this reason, they were integrated with semi-structured interviews with five service providers involved in the residence registration process. Moreover, it is difficult to generate official statistics by mapping the presence of homeless people and their characteristics; this is related to the hidden nature of the phenomenon, especially for homeless people who are not registered.

### About the interviews

The COVID-19 pandemic and its related restrictions did not allow us to meet with homeless people in person or arrange interviews with them via telephone or Skype. Interviews with service providers were conducted via Skype due to the lockdown in Italy between May and September 2020. Each interview lasted 1 h. We interviewed the primary contacts for the five services mostly involved in residence registration in Milan. These included:

- Unità Coordinamento delle Emergenze Sociali del Comune di Milano (Direzione Emergenze Sociali, Diritti e Inclusione), or Unit on Social Emergency, run by Milan’s City Hall (Department Social Emergency, Rights, and Inclusion), is part of the city’s Social Policy Department, which is devoted to supporting the welfare of citizens, particularly marginalized groups.^18^ They supervise all activities for homeless people in the city included in the *ResidenzaMi* project.
- CARITAS Ambrosiana is the body of the Catholic Church of Milan dedicated to bringing the theme of charity to parish communities and coordinating charitable activities in the city.^19^ They have a broad range of supporting activities for marginalized groups and people facing temporary difficulty.
- Casa della Carità, or House of Charity, is a foundation established in 2002 by the Catholic Church of Milan.^20^ It takes care of young people, adults, and families who face serious socio-economic difficulties.
- Servizio Sociale Professionale Territoriale (SSPT) Municipio IX, or Municipality IX’s Social Services Territorial Unit, provides specific work programs in the field of protection, reception, and social integration.
- Centro Aiuto Stazione Centrale (CASC), or Central Station Help Centre, is a low-threshold service based in Milan’s central train station; and
- Avvocato di Strada, or Street Lawyers, is a voluntary association that provides free legal advice and assistance to homeless citizens in 55 Italian cities.^21^

**Fig. 1:**
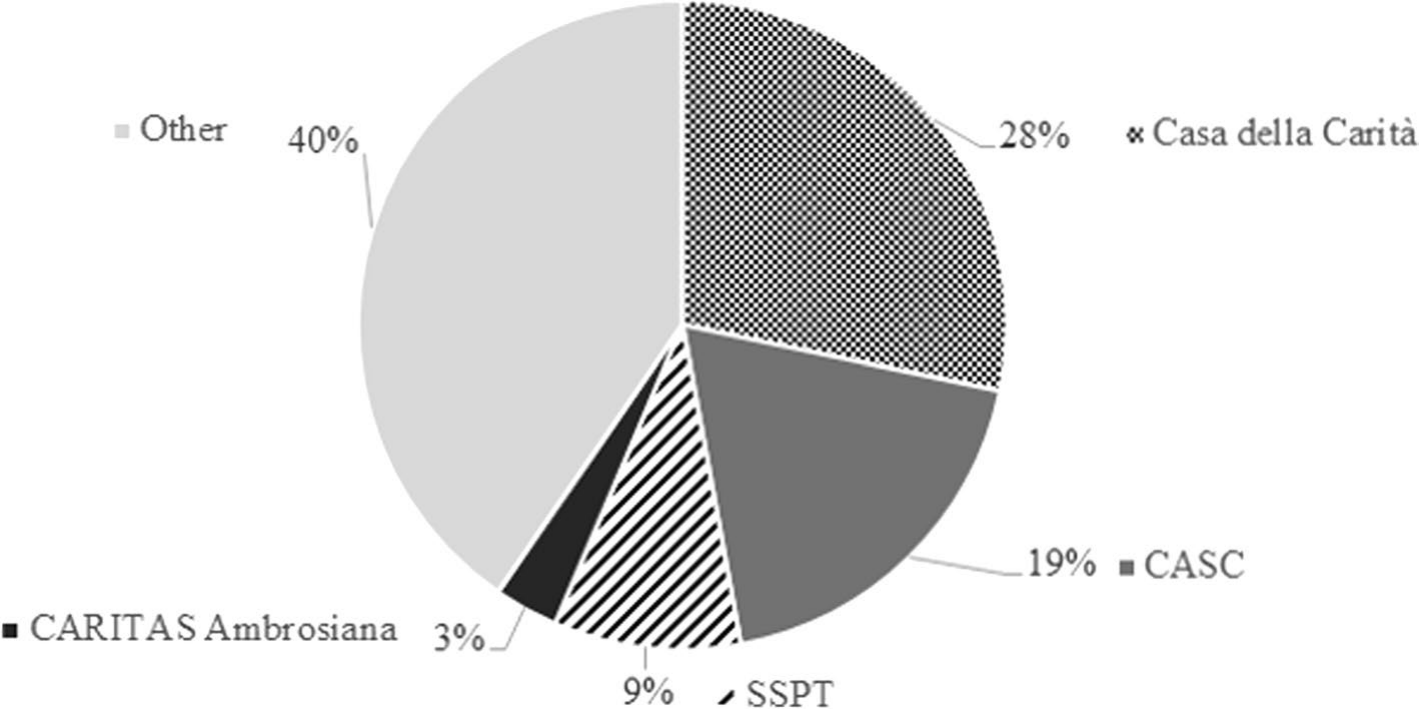
Sending services to *ResidenzaMi*. Source: *ResidenzaMi* register (2019). *n* = 1982. CASC = Centro Aiuto Stazione Centrale (CASC), or Central Station Help Centre; SSPT = Servizio Sociale Professionale Territoriale or Social Services Territorial Unit

Together, these services serve 60 percent of all homeless people involved in the *ResidenzaMi* project (Fig. 1). In 2019, there were approximately 1,982 applications for *ResidenzaMi*. About 47 percent of applications came from screening services (CASC and Casa della Carità, see Fig. 1). Three percent of the applications were managed by CARITAS Ambrosiana, while SSPTP referred about nine percent of the applications. The remaining 40 percent were managed by NGOs, parishes, and other entities from Milan’s third sector.

Interviews with service providers focused on the following topics:

- Activities for homeless people offered by the service and its specific involvement in residence registration;
- Changes in the registration system under *ResidenzaMi*;
- Description of main service users’ characteristics (homeless with or without residence certificates and differences between the two groups);
- Enabling and hindering factors in access to residence registration from the perspective of the service;
- Pros and cons of the registration system; and
- Social consequences of registration for homeless people.

Interviews were recorded and fully transcribed. They were analysed using MAXQDA-2020 according to the grounded theory approach (Strauss & Corbin, 1998). In particular, the interviews were coded to capture the different aspects related to the homeless population’s characteristics, the paths of access to residence registration for the homeless, and its effects. To protect participants’ identities, we made their responses anonymous and replaced the names of the people they mentioned with pseudonyms.

## Results

Our empirical results for Milan provide evidence on the importance of a residence certificate for improving the lives of homeless people and the crucial role services may play in improving the registration process. Our qualitative research findings supplement and enhance these results by providing further valuable insights from the perspective of service providers.

### The changing face of homelessness in Milan: who are the residence seekers?

It is interesting to note that, according to the service providers’ point of view, the Milan's homeless population asking for the residence certificate mostly includes people with a history of long-term unemployment and foreign nationals, who cannot renew their residence permits due to recent legal restrictions.

Interviews with service providers revealed that the characteristics of homeless people asking for residence registration continue to change over time. Although profiling homeless people is not simple, services providers agree with the perception that Milan’s homeless population does not match traditional stereotypes.

“Although most of them are so-called ‘traditional’ homeless people—single men living in extreme social marginality, an increasing number of them are families with dependent children.”*—*Respondent 1

About 36 percent of homeless people who applied for a residence certificate were Italian (Table 3), while the highest proportion included foreign nationals from outside the EU. “The irregular status of foreign nationals could be a reason for homelessness per se” (Respondent 2). Foreign nationals are usually people who come from Southern Italy, where they first arrive from their country of origin*. “*When they arrive in Italy, they often have a resident permit. Then they come to the north seeking a job, and they often live together in one apartment” (Respondent 3).

Some foreign nationals arrived in Italy without a residence permit (i.e. for work or family reasons, or to study) and then faced difficulties related to Italy’s immigration legislation.

“When they no longer have the possibility of renewing a residence permit, they easily fall into a cycle of difficulties, such as finding a regular job. They rely on precarious work situations, which makes it difficult to pay a regular rent or, in many cases, have a stable home.”*—*Respondent 6

One example comes from the story of Mohammed, described here by a service provider:

“He was one of the first people hosted here. He was born in 1941 and was an acrobat in the circus for about 20 years in Italy, where he arrived as a regular immigrant in 2000. He worked in circuses in Italy and Europe with a residence permit, but he didn’t think about his future. When he turned 59, the circus told him: ‘It was nice, but we don't need you anymore, it is over!' He had paid some contributions for the pension off and on. When he stopped working, he lost his residence permit, and he had no savings or a pension. He started drinking and living on the street before we took him in, and he died here of a heart attack. He was one of us, in short.”*—*Respondent 3

Traditional homeless people who “usually show anti-social behaviours, severe mental disorders, and alcohol addiction” are a residual part of the group of individuals using low-threshold services and asking for the residence certificate, which is mainly composed of those who “usually have a low level of education and a long experience of unemployment” (Respondent 2). Respondents noted that there is generally a progressive path of self-deterioration as “it is rare to meet a person who became homeless from one day to the next” (Respondent 2). The process is slow. It may start with repeated periods of unemployment, often followed by a disruption in family ties and separation, which leads men out of the house and onto the street. However, before arriving on the street, some “reached out to their peer group first, sleeping on their sofa or in their car. Very often, they start to abuse alcohol as a coping mechanism” (Respondent 2)*.* Before accessing social services and starting the process of residence registration, homeless people draw from their own resources (money, family, friends, etc.) and only ask for help from low-threshold services as a last step. As a result, service providers perceive that “those who arrive are surely already ‘selected’ and will have a new chance of exiting from homelessness” (Respondent 2). Heroin addiction, however, has re-emerged in Italy, “especially among adolescents, who often leave their family home to live on the street” (Respondent 2).

Finally, low-threshold service providers have noticed that those asking for a residence certificate are also people who cannot show a rental or ownership address, such as abusive occupants or irregular tenants who do not necessarily live on the street or sleep in public dormitories.

“They may even be the 85 percent who apply for a residence certificate. They might be a normal employee—with precarious and low-income jobs—who cannot afford regular rent but who do not belong to the homeless group. This group does not necessarily use low-threshold and first aid services, but they might need it, for example, for a discount for their kid’s school canteen or to get a family doctor.”*—*Respondent 2

### Barriers to residence registration

According to interviews with service providers, there are three main barriers to accessing a residence certificate: cognitive factors, fear and mistrust, and social stigma. For foreign born individuals, residence permit is also a prerequisite to residence registration.

### Cognitive factors

Service providers report how homeless people often do not know that the possibility of being registered exists. Often, they “are so tested by life that they do not even rise from where they are” (Respondent 3). Service providers noted that homeless people do not know or understand the real importance of having a residence:

“Most of them think their residence is the place where they live. They think they don’t need it, since cities like Milan offer a lot of services—mobile units that take you to eat, dress, visit canteens, or access the surgeries of the ‘Opera San Francesco’ where you can have all the visits and medicine for free.”—Respondent 2

Not everyone can grasp the fundamental need for documents as tools for transformation. *“*Although they may have the documents, they can lose them, and so they have to redo the process again and again” (Respondent 1). Indeed, to apply for a residence certificate “[…] you have to get active and go to a public office, in an institutional context. You have to choose to do things, and so the choice is to take that first step. You have to want change” (Respondent 1).

Some cognitive factors push homeless people to act and transform their lives; other times, people take action because of a relationship.

### Fear and mistrust

Another barrier to registration relates to psychological aspects, such as discomfort and mistrust, that homeless people may experience. As one service provider noted, “They don’t consider recognition by the City to be important, as they feel abandoned by the society” (Respondent 5)*.* Service providers expressed widespread feelings of mistrust, “especially towards municipal social services, as homeless people think they don’t do enough to help them” (Respondent 2). Foreign nationals may avoid residence registration, fearing they will not be allowed to stay in Italy and face deportation when their residence permit expires.

Therefore, “a long relationship” with services is the only way to “inspire trust and lead to a residence certificate” (Respondent 3). Service providers’ work is based on relationships. For example:

“Being able to solve problems, such as legal situations, leads users to change their perspective about society, to understand that problems can be solved, that difficult situations can be overcome, that not everything is lost but that there is a possibility to get your hands on the problems, to face them with someone who can help.”*—*Respondent 5

For service providers, building a relationship is not always easy and it is often not clear where the service’s efforts will lead; however, respondents acknowledged that support could help people to leave the streets.

“Luigi had a job in Germany, but he had failed. He spent some time in a jail, then he arrived in Italy and he was with us for a month or two. Then he disappeared, and we never saw him again. After a few months, we got a postcard from Dubai from him saying ‘Thanks for what you did.’”—Respondent 3

### Social stigma

Finally, social stigma can destroy relationships and distance homeless people from those who could help or give them support (Respondent 5). When homeless people come from safe, normal conditions but unexpectedly find themselves in situations of absolute poverty and indigence, “there is then a social stigma that characterizes their relationships, which leads them to distance themselves from friends and family members who no longer want to give support. They find themselves alone and lonely, which is probably the real reason that leads them to homelessness” (Respondent 5). Social stigma enhances loneliness and may be a major barrier toward residence registration. Indeed, homeless people may experience negative judgement from other people—as though they were to blame for their homelessness. This may encourage them to avoid social relationships and become as invisible as possible. Fewer social interactions limit their chances of building trusting relationships with service providers and starting the process of residence registration. In addition to facing stigma about being homeless and foreign, foreign nationals in Italy are also often single individuals arrived in Italy alone and lack support from their families of origin living abroad.

### Practical advantages of a residence certificate

Despite the barriers, service providers recognized that beyond a desire to be registered, homeless people also seek residence registration as an opportunity to access material benefits.

### Economic and housing benefits

The economic benefits of registration include *reddito di cittadinanza,* or citizenship income, social security services, and a home.^22^ Indeed, to apply for inclusive income support and *casa popolare,* or public housing in Milan, a person must have been a resident of Lombardy region, of which Milan is the capital.^23^

“Since the job market is completely closed to homeless people—who might have mental diseases, addictions, or who might behave antisocially—they survive thanks to assistance measures. This is their main motivation for applying for a residence certificate, as a way to escape from homelessness.”—Respondent 2

### Free legal assistance

Based on the experience of service providers, homeless people also apply for a residence certificate to obtain free legal assistance in case they need a lower rent*,* which is particularly important for foreign nationals.

“Following the adoption of the Security Decree, there was a significant increase in users who visited our branches to deal with this type of issue. They required support both in dealing with illegitimate expulsion measures, or even trivial delays in the execution, resolution, or conclusion of related immigration procedures. Our service involved offering practical support by accompanying them to the proper authorities, the police, or the prefectures, as the case may be, to try to unblock regularization procedures.”*—*Respondent 5

### Ability to receive mail

In addition, we found that the ability to receive mail plays an important role in the desire to obtain a residence certificate, among other things.

“Receiving mail is something we take for granted, although it is fundamental to maintaining relational, work, and family ties. Without an address to receive mail, it is impossible to receive institutional correspondence, such as a new document, a copy of an identity document, a ballot, or a health card.”*—*Respondent 5

Correspondence is an important topic, especially for those with disabilities because “[…] receiving pensions and economic support for a disability requires a residence because they require documented proof about interactions with officials, visits, and examinations” (Respondent 5).

In most cases, there is a specific event in a person’s life that reveals the urgency of being registered. More specifically, it is likely that people discover the concrete advantages linked to a residence certificate in a condition of need, such as a serious illness. Under normal conditions, “they do not really need a residence certificate to access health care” (Respondent 4) as first aid and emergency services are guaranteed. However, they need a residence certificate to get a family doctor who can prescribe medication.

### Overarching benefits

Residence registration may be the first formal step toward better living conditions for homeless people. It is the key path toward several services and opportunities that may help people overcome the hardships of living in the street. However, as we have seen, it is not only a bureaucratic procedure, but much more an existential path. “When one is on the street, it’s another world. On one hand, he has finished fighting. On the other hand, he needs endless strength to fight, because living on the street is particularly hard” (Respondent 3). Service providers build relationships using professionalism and empathy to gradually follow and support people, as the process may take several meetings.

“You walk him slowly through the residence registration. You make him see another possible world, another perspective, another way to enrich his life. For example, he may realize that income can be interesting, or that registration is important because then he will have access to a doctor. It's a different worldview.”—Respondent 3

### Impact of COVID-19 on residence registration and services

The outbreak of the SARS-CoV-2 virus and the associated COVID-19 pandemic quickly spread across the globe, and several countries adopted lockdown and physical distancing rules. Italy, and Milan in particular, were strongly affected by the pandemic in terms of cases and death rates.

The pandemic made life even more difficult for the homeless population. Many services for homeless people were closed due to restrictions imposed by the government and their fragile routine was destroyed. In such situations, interpersonal relationships may deteriorate, create distrust in people or institutions, and cause people to feel abandoned by society. Although new services were developed in Milan to cope with the unexpected situation, even low-threshold services had trouble tracking and supporting homeless people during lockdown, with people forced to stay at home.

Although the pandemic temporarily blocked the registration process, services never stopped. Instead, they adapted to the emergency. For example,

“Only half of the public showers have been opened, and new a health service has been provided for temperature detection. We had to put chemical toilets on the streets, because people can no longer use the bathrooms in bars. In addition, municipal services have increased food distribution using mobile units, although the standard procedure was to lead people to canteens and not feed them on the street. They used a building with 48 apartments to help homeless people with symptoms quarantine.”—Respondent 1

Other services switched to an appointment model or shifted their work to remote assistance since they could not receive homeless people physically. However, the biggest problem was the dispersion of homeless people and the loss of contacts among them. Physical distancing measures, including closing dormitories or day centres, spread homeless people out over the national territory (Respondent 5).

“It was dramatic because it led to detachment among homeless people. The protection network is mainly linked to associations, but also to residents in the neighbourhoods where they usually spend the night. COVID-19 led to the disintegration of these networks. Many homeless people were displaced and got lost. Where we once had stable places with fixed hours that allowed people to freely access our services, with the lockdown we were no longer able to reach them.”—Respondent 5

Moreover, the COVID-19 pandemic revealed the need for greater social and health services integration, especially for people living on the streets, who face many difficulties in taking medication, accessing medical care, and attending medical surgeries (Respondent 1). Low-threshold services in Milan also had to deal with cases of positive individuals with symptoms, for whom it was difficult to understand and respect the quarantine and movement restrictions. For example, “many people who tested positive for COVID-19 were young riders who make a living delivering meals, foreign nationals, and former refugee applicants” (Respondent 2). Another service provider added, “they are scared. They still struggle to understand why we need to do these things, how to use the devices, or why hygiene is such an issue” (Respondent 1).

Overall, the greatest risk was losing people with whom the service providers had built solid relationships. For example, one respondent noted:

“Leonardo is Italian. He is 60 years old and has been in our dormitory for 15 years. With the lockdown, he was not able to stay with us, without going out. After 15 years, we lost him. We wondered if he was dead. We looked for him also at the Musocco cemetery, at Campo 87, where there are hundreds of graves. We made an announcement to find him. Finally, we found him at the hospital. He was infected with COVID-19 and he had been hospitalized for a month.”—Respondent 3

## Lessons learned

This study sheds important light on the successes realized by residence registration for homeless people, in particular, through the *ResidenzaMi* program in Milan. The following key lessons may help guide future research on the topic:

**Overcoming the obstacles to accessing a residence certificate requires a systemic approach**. One of the crucial weaknesses of the Italian system is the lack of consistency in the procedures to access a residence certificate from city to city in Italy. Milan’s proactive approach to helping homeless people access residence registration can serve as a template for other Italian cities.

**Having an address to receive official notices from public authorities is crucial to giving homeless people access to services and have ID card**. Service organizations that provide a residence address for homeless people and support them in the registration process report how they quickly become a sort of “post office” that deals with hundreds of letters each day. This basic but fundamental service allows homeless people to interact with government services to physically receive official messages or documents, such as the health card, but also the marriage certificate or the notification of support requests (e.g., economic and job incentives, etc.). The residence certificate is also a prerequisite for the ID card that is proof of legal identity essential to obtain other documents, functional to access services and rights.

**Relationships have an important impact on residence registration**. Although the *ResidenzaMi* project was thought to eliminate all the obstacles and barriers to civil registration, many homeless people are still not registered. This is partly due to factors such as the recent implementation of the project and the COVID-19 pandemic restrictions. However, residence registration is often a process that can only start when a homeless person and a professional in charge of the registration service develop a trusting relationship. This trust allows homeless people to see institutions and offices as allies rather than enemies.

**Foreign nationals face specific challenges to residence registration**. Residence registration is higher among foreign nationals, who face specific challenges to residence registration. Foreign nationals living in Italy face language and cultural barriers and bureaucratic challenges (for example, having birth certificates that are not recognized by Italian authorities, or needing a residence permit to get a residence certificate but being unable renew their residence permit without a residence certificate). Foreign nationals also lack social and economic support from a family network in Italy. Immigration policies and residence registration should consider and address difficulties experienced by foreign nationals in obtaining and maintaining their legal identities due to the denial, invalidation, and dispossession of official documentation (Cheong & Baltazar, 2021). Further research is needed to consider the particular requirements of foreign nationals and ease the specific difficulties they face in obtaining a residence permit.

**More information on residence registration is required at the national level to improve service design and delivery**. Since contextual factors related to the residence certificate are missing in the national surveys, more information should be collected to facilitate service design and improve dedicated services.

**Future research should examine the requirements for scaling up the ResidenzaMi project in other cities and contexts**. Italy's disjointed (or heterogenous) approach to serving homeless populations, as in other countries, would benefit from an evidence-based approach to providing better life opportunities and outcomes for the homeless population through residence registration support.

## Conclusion

This paper aimed to gain a better understanding of the access paths to residence registration for the homeless population, in this case, from the perspective of low-threshold service providers. Our empirical results on Milan provide evidence on how important a resident certificate is to the lives of homeless people and the critical role that services may have in favouring the registration process.

Homeless people experience extreme poverty and a severe lack of material, emotional, and relational resources (Johnson et al., 2005; LaGory et al., 2001; Lee et al., 2010; Meanwell, 2012). Having access to services is fundamental to normalizing all other life spheres and escaping homelessness (Batterham, 2019; Cortese, 2017; Fowler et al., 2019; Ministero del Lavoro e delle Politiche Sociali, 2015). Thus, access to social services is crucial to alleviate this extreme situation.

Overall, our findings suggest that a residence certificate is a key factor for accessing services such as public housing and a family doctor, which might be the first step and key access point for many opportunities to improve the living conditions of homeless people in Italy. Moreover, our analyses have indirectly shown that to fully enjoy rights and to access health and social services, people need to be legally identified by the State. The residence certificate is a prerequisite for the ID card that is a proof of legal identity enabling individuals to access such services.

This study sheds light on the process of residence registration for homeless people in Milan by identifying its main barriers and enabling factors. This is a complex process where national law, projects by local administrations, NGO activities, and interpersonal dynamics interact (Batterham, 2019; Meanwell, 2012).

Interviews with service providers identified that Milan offers many low-threshold and first aid services to the homeless population, such as public showers, canteens, or dormitories. However, a residence certificate is required to access economic support measures, request public housing, receive local social services, and access health care.

This study had some limitations due to the lack of national data related to the residence certificate. Available ISTAT national surveys rely on a representative national sample of homeless people. Although they investigate several dimensions, they lack detailed information about the life course events of homeless people and the paths they use to access identity documents and residence registration. Nonetheless, *ResidenzaMi* can be used as an example of the effective provision of residence certificates to the homeless population. Finally, due to the COVID-19 pandemic and restrictions on personal mobility, it was not possible to interview homeless people in this study. However, since their voices are fundamental to this research, as a next step, research should include their point of view on residence registration, motivations, challenges, and the effects of registration or non-registration on their lives.

Evidence suggests the need to further explore the critical role of residence registration in improving the lives of homeless people in Italy and other countries. Indeed, increased barriers to legal identity access are not just an Italian phenomenon (Cheong and Balatazar 2021). Future research should consider the multidimensional factors involved in the residence registration process for homeless people at the country level. Although more information is needed, this study demonstrates how a residence certificate is essential to improving the lives of homeless people.

This paper has shown that access to proof of legal identity is critical to helping homeless people in Italy access social and economic support from the State. However, homeless people face particular challenges in proving their legal identity and accessing residence registration, both of which are required to access social services. Foreign nationals make up approximately 75 percent of the homeless population in Milan. This suggests that this subgroup faces additional challenges in terms of cognitive factors, fear, mistrust, and social stigma. The lack of residence registration for foreign nationals increases their vulnerability, promotes socio-economic exclusion, and prevents them from fully participating in and contributing to society. Further synthesis of qualitative research and survey and administrative data can strengthen the evidence base for improved policy and practice in support of such vulnerable subpopulations. To do this effectively, future ISTAT surveys and administrative data should include and monitor information about life course events of homeless people and the paths they use to access residence registration. Considering the barriers to legal identity that homeless people face can help create inclusive societies, however, it is an area of social science that requires further study.

## Data Availability

The datasets used and/or analysed during the current study are available from the corresponding author on reasonable request.

## Footnotes

1 Italy’s homeless population was estimated at 50,724 people (ISTAT, 2015) and the national representative sample consists of 4726 people. Data are drawn from the ISTAT (Year 2014) Detection of Extreme Poverty survey. Processing was carried out at the ISTAT Laboratory for the Analysis of Elementary Data in compliance with legislation on statistical law enforcement and the protection of personal data.

2 Residence registration is required in several EU countries where people must meet residence-based conditions to access services (Bruzelius, 2019).

3 Once the individual has obtained the residence registration, he/she can ask for the ID card at the City Hall following the standard national procedure.

4 Birth and marriage certificates are not foundational ID systems, such as national ID card. Moreover, residence certificate is a prerequisite for marriage and civil union, but not for birth registration.

5 As outlined in national Law no. 30/2007 and Law no. 286/1998.

6 For further information, see the website: https://www.fiopsd.org/vademecum-residenza-per-i-senza-dimora/.

7 77 percent of the Milan’s homeless population stays in dormitories vs 52 percent of the Roma’s one (Fondazione Rodolfo de Benedetti, 2018).

8 This information was collected using a Street and Shelter Night approach to count all of Milan’s homeless people in a single night in a whole metropolitan area. This approach minimizes the risk of counting the same person twice and includes all people who reside in cars, parks, sidewalks, abandoned buildings, emergency shelters, disused areas, shacks, and slums on a given night (Braga & Corno, 2011).

9 Notice that only data coming from the same source can be compared. Namely, those from fRDB 2008, 2013 and 2018.

10 PON INCLUSIONE Avviso 4. https://www.poninclusione.lavoro.gov.it/.

11 Translation of the Italian term, *miseria ladra.* https://www.miserialadra.it/

12 Before 2019, *Casa della Carità* were used to provide an address to about 2,000 individuals per year.

13 Given that not all of the city’s halls agreed to open a desk for the residence registration, some have been clustered to cover the entire territory.

14 Although the service was born for homeless individuals, the problem of a lack of residence concerns a much bigger group of persons. Thus, *ResidenzaMi* decided to expand the definition of eligibility for applicants, including abusive tenants, for example.

15 To make the process as homogeneous as possible, all social services and parishes that registered homeless people at their own address before 2019 are now asked to converge the residences of homeless people into the *ResidenzaMi*.

16 For details of the procedure, see https://www.comune.milano.it/servizi/fare-la-carta-di-identita.

17 This statistic is in line with the number of single men in relation to the total homeless population without a residence certificate in Milan, which is about 72 percent. Elaboration from the ISTAT (Year 2014) Detection of Extreme Poverty survey. Not shown in table).

18 https://www.comunemilano.it.

19 https://www.caritasambrosiana.it.

20 https://www.casadellacarita.org.

21 https://www.avvocatodistrada.it.

22 Citizenship income was introduced by legal Decree no. 4 of 2019. It provides a basic income and is an active labour policy designed to fight poverty, inequality, and social exclusion.

23 Public housing (government-subsidized housing) includes accommodations owned exclusively by the municipality that can be rented to individuals or families who find themselves in difficult economic conditions.
